# Regulation and Roles of Urocortins in the Vascular System

**DOI:** 10.1155/2012/873723

**Published:** 2012-05-13

**Authors:** Kazunori Kageyama, Ken Teui, Naoki Tamasawa, Toshihiro Suda

**Affiliations:** Department of Endocrinology and Metabolism, Hirosaki University Graduate School of Medicine, 5 Zaifu-cho, Hirosaki, Aomori 036-8562, Japan

## Abstract

Urocortins (Ucns) are members of the corticotropin-releasing factor (CRF) family of peptides. Ucns would have potent effects on the cardiovascular system via the CRF receptor type 2 (CRF_2_ receptor). Regulation and roles of each Ucn have been determined in the vascular system. Ucns have more potent vasodilatory effects than CRF. Human umbilical vein endothelial cells (HUVECs) express Ucns1-3 mRNAs, and the receptor, CRF_2a_ receptor mRNA. Ucns1-3 mRNA levels are differentially regulated in HUVECs. Differential regulation of Ucns may suggest differential roles of those in HUVECs. Ucn1 and Ucn2 have strong effects on interleukin (IL)-6 gene expression and secretion in rat aortic smooth muscle A7r5 cells. The increase that we observed in IL-6 levels following Ucn treatment of A7r5 cells suggests that smooth muscle cells may be a source of IL-6 secretion under physiological stress conditions. Ucns are important and unique modulators of vascular smooth muscle cells and act directly or indirectly as autocrine and paracrine factors in the vascular system.

## 1. Introduction

Corticotropin-releasing factor (CRF) plays a central role in controlling the hypothalamic-pituitary-adrenal (HPA) axis during stress [1]. Urocortins (Ucns) are also members of the CRF family of peptides. Three Ucns have been found in mammals. Ucn1 is a 40-amino-acid peptide cloned from the Edinger-Westphal nucleus [2], and Ucn2 and Ucn3 are identified in the human genome data base and in mouse genomic DNA, respectively [3–5]. Ucn1 and/or Ucn2 is expressed in the heart, vascular, and peripheral blood cells [3–8], while the expression of Ucn3 also has been reported in the human cardiovascular system [9]. The Ucns have been demonstrated to play important modulatory roles in various tissues including the brain, immune system, cardiovascular system and gastrointestinal system, and may be important in the various stages of atherosclerosis development [10].

**Figure 1:**
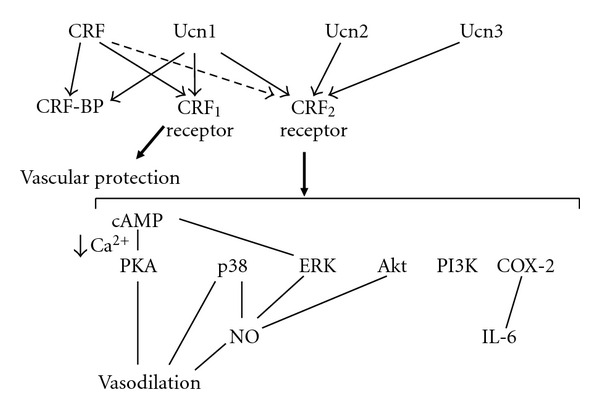
Proposed signaling mechanisms of Ucns and CRF receptors in the vascular system. CRF-BP, CRF-binding protein.

The actions of the CRF family peptides are mediated by at least two distinct G protein-coupled receptors, namely, the CRF receptor type 1 (CRF_1_ receptor) [11–13] and CRF receptor type 2 (CRF_2_ receptor) [14–16]. These two receptors share 69% amino acid homology [17], but have different tissue distributions and pharmacological properties with respect to ligands [10]. CRF_1_ receptor is the major subtype responsible for regulating synthesis and secretion of adrenocorticotropic hormone (ACTH) in the pituitary corticotrophs [18], whereas CRF_2_ receptor with splice variants is found in the brain and in peripheral sites including the cardiac myocytes and vascular smooth muscles [19, 20]. CRF has higher affinity for the CRF_1_ receptor than for the CRF_2_ receptor (Figure 1). Ucn1 binds to both the CRF_1_ and CRF_2_ receptors, while Ucn2 and Ucn3 are highly selective for the CRF_2_ receptor, with little affinity for the CRF_1_ receptor (Figure 1) [2, 4, 5].

CRF_2b_ receptor is a member of the Class B heptahelical G protein-coupled receptors. This receptor is positively coupled to adenylate cyclase and is bound preferentially by the CRF-related peptides, Ucns. In the rodent, CRF_2b_ receptor messenger RNA (mRNA) is expressed in the cardiovascular system, where its levels can be modulated by Ucns [24]. Ribonuclease protection assays show that A7r5 cells express the CRF_2b_ receptor subtype, which have two isoforms differing in one codon at the junction of exons 3 and 4 [25]. Ucn induces accumulation of intracellular cAMP via CRF_2b_ receptor. Ucn induces intracellular cAMP to downregulate CRF_2b_ receptor mRNA expression in A7r5 cells [25]. CRF_2_ ligands or dexamethasone reduces CRF_2b_ receptor mRNA levels [24]. In addition, a variety of cytokines also reduce CRF_2_ mRNA expression [24]. The multifactorial regulation of CRF_2_ mRNA expression in the cardiovascular system may serve to limit the inotropic and chronotropic effects of Ucns during prolonged physical or immune challenge.

## 2. Expression and Regulation of Ucns in the Vascular System

**Figure 2:**
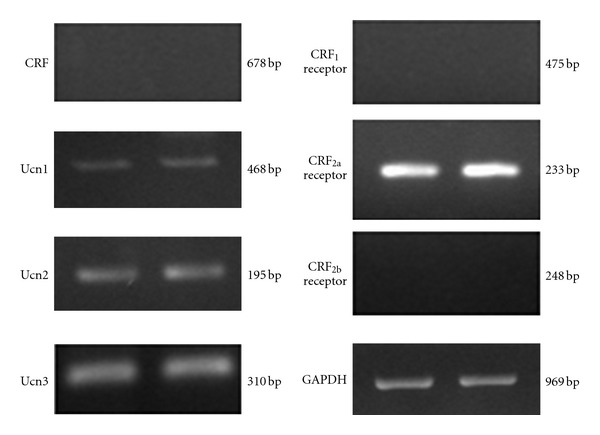
Expression of Ucns and CRF receptors mRNA in HUVECs. A representative image of RT-PCR for Ucns and CRF receptors mRNA in HUVECs. Reproduction from [21] with permission of the publisher. Copyright 2009, Elsevier.

Human umbilical vein endothelial cells (HUVECs) express Ucns 1–3 mRNAs, and the receptor, CRF_2a_ receptor mRNA, suggesting an endogenous role of each Ucn via the CRF_2a_ receptor in HUVECs (Figure 2) [21]. Endogenous Ucn in the system might act in an autocrine or paracrine manner [26]. Endothelial Ucn1, upregulated by inflammatory cytokines and pitavastatin, suppresses reactive oxygen species production in endothelial cells [7]. The data suggest that endothelial Ucn1 has potent antioxidative properties [7]. Lipopolysaccharide (LPS) decreases Ucn1 mRNA levels, while it increases Ucn2 and Ucn3 mRNA levels in HUVECs [21]. LPS would regulate Ucns gene expression levels directly through Toll-like receptors. After immune stimulation, tumor necrosis factor (TNF)-*α*, interleukin (IL)-1, and IL-6, are elevated in the systemic circulation. These cytokines also increase the activity of the HPA axis, resulting in the release of additional ACTH and corticosterone [27, 28]. Among them, IL-1*β* is a pleiotropic cytokine with a variety of biological activities. IL-1*β* decreases Ucn1 and Ucn2 mRNA levels, while it increases Ucn3 mRNA levels in HUVECs [21]. These data are consistent with changes in mRNA levels of Ucn1 and Ucn3 following LPS. Therefore, IL-1 and LPS may contribute cooperatively to regulate the levels of Ucn1 and Ucn3 mRNA in the vascular cells.

Forskolin stimulates adenylate cyclase and then intracellular cAMP in HUVECs. Norepinephrine, prostacyclin, and adiponectin may be candidates for natural ligands to activate cAMP pathway in HUVECs [29–31]. These peptides or hormones can regulate Ucns via the cAMP production in HUVECs, although this association remains speculate. Ucns also regulate own or other Ucns in an autocrine or paracrine manner via the cAMP pathway. In fact, forskolin increases Ucn1 mRNA levels, while it decreases Ucn2 and Ucn3 mRNA levels in HUVECs. In both the mouse and human Ucn promoters, there is a consensus cAMP response element (CRE) site, which has been shown to mediate the regulation of Ucn expression by cAMP [32]. The CRE binding protein via the protein kinase A (PKA) pathway may be involved in this pathway. Ucn2 and Ucn3 mRNA levels are decreased presumably due to increase in mRNA degradation or decrease in the mRNA synthesis. Differential regulation of Ucns1-3 mRNA may suggest differential roles of those in HUVECs.

## 3. Roles and Action of Urocortins in the Vascular System

**Figure 3:**
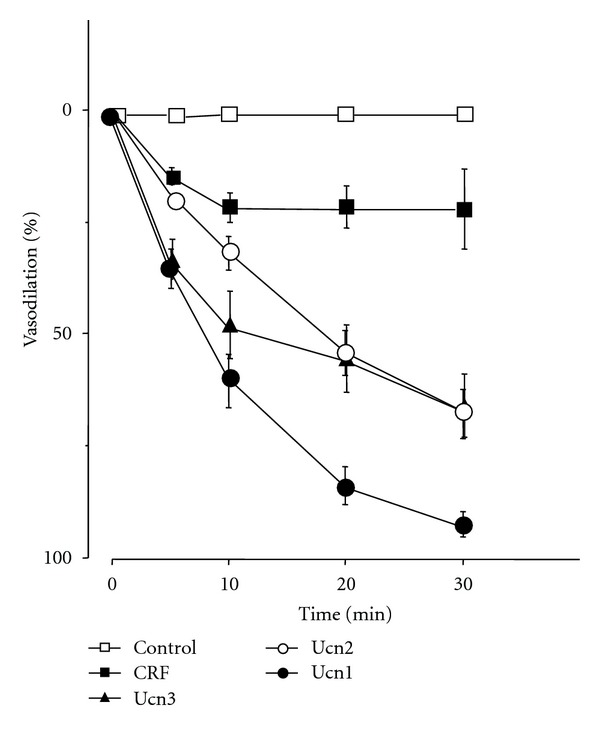
Effects of CRF and Ucns on vasodilation. Rat aortae were incubated with medium alone (control) or with medium containing 1 *μ*M of CRF, Ucn1, Ucn2, or Ucn3 (*n* = 5). Statistical analyses were performed using two-way ANOVA, followed by Scheffe's F post hoc test. Reproduction from [22] with permission of the publisher. Copyright 2003, Lippincott Wiliams & Wilkins.

Recent studies have shown the potent effects of Ucns on the cardiovascular system. Taken together with the expression of Ucns and the receptor, endogenous Ucns have a physiological role in the cardiovascular system. Vasodilatory effects of Ucns have been demonstrated in rat tail and basilar arteries [33, 34]. Ucn1 produces vasodilation via the adenylate cyclase and PKA pathway [34]. Furthermore, Ucn1 and Ucn2 have more potent vasodilatory and cardiac inotropic effects than CRF, with a greater potential to increase coronary blood flow and reduce overall blood pressure [34, 35]. In our previous study, Ucns1-3 were more potent vasodilators than CRF in a rat thoracic aorta model (Figure 3) [22]. Ucns contribute to vasodilation via p38 mitogen-activated protein (MAP) kinase and PKA pathways (Figure 1). In vascular smooth muscle cells, stimulation of CRF_2_ receptors results in increased cAMP accumulation via activation of adenylate cyclase [25]. It is at least possible that increased cAMP levels contribute to vasorelaxant responses, although the role of cGMP remains unclear. Ucns exert their vasorelaxant effects via G *α* s-cAMP-PKA signaling, leading to downregulation of the phospholipase C*β*-inositol 1, 4, 5-triphosphatase-Ca^2+^ signaling pathway (Figure 1) [36]. The Ucns-induced endothelium-dependent relaxation of rat coronary arteries would be also attributable to endothelial nitric oxide (NO) (Figure 1). Ucn2 induces NO production through cAMP-dependent and Ca^2+^-related phosphorylation of extracellular signal-related kinases (ERKs), Akt, and p38 pathways in aortic endothelial cells (Figure 1) [37]. In addition, we reported a first case of multiple endocrine neoplasia type II without hypertension, accompanied by thyroid medullary carcinoma and pheochromocytomas expressing CRF, Ucn1, and Ucn3 [38]. This case highlights that CRF and Ucn1 secreted from the adrenal pheochromocytomas and thyroid tumor might induce vasodilation.

Ucn2 significantly reduces blood pressure in hypertensive rats without affecting heart rate [39]. Long-term Ucn2 treatment in hypertensive rats induces sustained blood pressure reduction and diminishes the development of hypertension-induced left ventricular hypertrophy and the deterioration of left ventricular contractile function [39]. CRF_2_ receptor expression levels are preserved, despite chronic stimulation by Ucn2. Ucn also may play a role in vascular remodeling [40]. Long-term Ucn treatment not only has hypotensive effects but also may inhibit development of vascular remodeling in mesenteric arteries in spontaneously hypertensive rats [40]. Together, CRF_2_ receptor stimulation by Ucn2 may represent a novel approach to the treatment of arterial hypertension.

Endothelial Ucn1 has potent antioxidative properties. Treatment with pitavastatin (2 mg/day) for 4 weeks increases the serum Ucn1 level from 11.0 ± 6.5 to 16.4 ± 7.3 ng/mL in healthy subjects [7]. Thus, endothelial Ucn1 may protect cardiomyocytes in inflammatory lesions. The selective blockade of CRF receptors expressed in human aortic endothelial cells also indicates that CRF_1_ receptor signaling mainly exerts anti-inflammatory actions [41]. The beneficial action of pitavastatin may be, in part, exerted via CRF_1_ receptor (Figure 1). 

**Figure 4:**
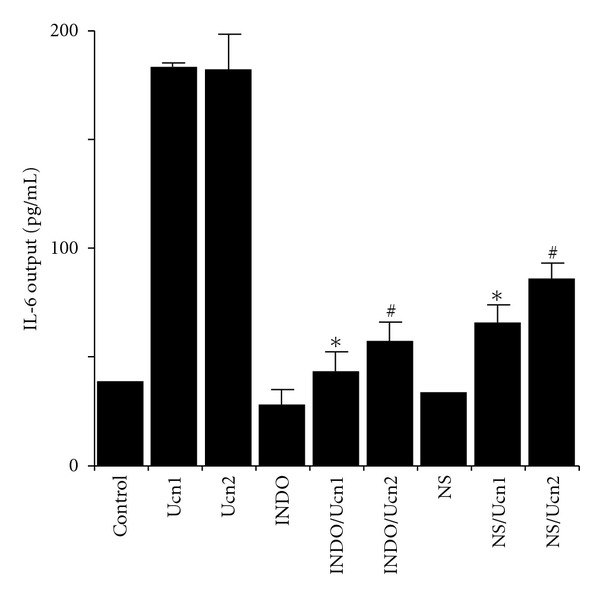
Effects of cyclooxygenase inhibitors on Ucn1- or Ucn2-induced IL-6 output in A7r5 aortic smooth muscle cells. Cells were treated in triplicate, with the mean of three independent experiments (an average in triplicate was considered *n* = 1; three experiments *n* = 3) shown in figures. Statistical analyses were performed using one-way ANOVA, followed by Scheffe's F post hoc test. Cells were preincubated with medium containing indomethacin (INDO), NS-398 (NS), or vehicle for 30 min then incubated for 48 h with medium containing 100 nM Ucn1, Ucn2, or vehicle. *P < 0.0005 (compared with only Ucn1). ^#^ *P* < 0.0005 (compared with only Ucn2). Reproduction from [23] with permission of the publisher. Copyright 2006, The Endocrine Society.

Ucn1 and Ucn2 have strong effects on IL-6 gene expression and secretion in rat aortic smooth muscle A7r5 cells [23]. Cyclooxygenase-2 (COX-2) pathway is involved downstream in regulation of Ucn-increased IL-6 gene expression and IL-6 secretion (Figure 4) [23]. The increase that we observed in IL-6 levels following Ucn treatment of A7r5 cells suggests that smooth muscle cells may be a source of IL-6 secretion under physiological stress conditions. Increased IL-6 protein levels would be expected to modify both humoral and cellular immunity [42]. In addition, IL-6 is able to stimulate ACTH and glucocorticoids secretion [43]. This combination of actions implies that increased IL-6 levels may have direct and indirect effects on the immune and other stress modulations. We have demonstrated previously that Ucn directly downregulates CRF_2b_ receptor mRNA levels [24]. Because cytokines such as IL-1 and IL-6 both decrease CRF_2b_ receptor mRNA expression [24, 44], it is possible that Ucn and IL-6 contribute cooperatively to regulate the levels of CRF_2b_ receptor mRNA in vascular cells. IL-6 may act as an autocrine and paracrine factor in the vessel wall.

Ucns have been suggested to have roles in regulation of blood pressure and in the pathophysiology of cardiovascular disease. Richards's group reports that plasma Ucn1 in normal control subjects at 7.2 ± 2.9 pM is significantly lower than levels recorded in the non-heart-failure symptomatic patients (11.1 ± 3.2 pM) [45]. Elevated plasma Ucn1 in human heart failure reflects a beneficial compensatory response to this condition. Serum Ucn2 levels are elevated in mild-to-moderate systolic dysfunction (12.8 ± 3.6  versus 10.4 ± 3.9 pg/mL, resp.) [46]. The studies by Rademaker et al. suggest that Ucn2 or Ucn3 may have therapeutic potential in patients with heart failure [47, 48]. Adjunct Ucn2 therapy with diuretics in heart failure is beneficial, because Ucn2 administration induces sustained improvements in hemodynamics and renal function, in association with inhibition of multiple vasoconstrictor/volume-retaining systems [49]. These findings support the therapeutic potential for Ucn2 in heart failure [50]. Ucn3 induces potent inhibition of sympathetic traffic directed toward the heart [51].

Ucn1 is a cardioprotective peptide and is also involved in cardiac hypertrophy. Ucn1-induced cardiomyocytes hypertrophy is associated with regulation of GSK-3*β*, a pivotal kinase involved in cardiac hypertrophy, in a phosphatidyl-inositol-3-kinase- (PI3K-) dependent manner [52]. The expression of endogenous cardiac Ucns is increased by *in vitro* ischemia-reperfusion damage, and the addiction of exogenous Ucns is associated with reduction of myocardial cell death during ischemia-reperfusion damage [53]. Therefore, Ucns have a significant protection against myocardial ischaemia/reperfusion injury [54, 55].

## 4. Conclusion

HUVECs express Ucn1, Ucn2, and Ucn3 mRNAs and CRF_2a_ receptor mRNA. Differential regulation and roles of Ucns1-3 mRNA are suggested in HUVECs. Ucn1 and 2 stimulate IL-6 gene transcription and secretion via CRF_2_ receptor activity in A7r5 aortic smooth muscle cells. Ucns are important and unique modulators of vascular smooth muscle cells and act directly or indirectly as autocrine and paracrine factors in the vascular system.
